# Synthesis, Characterization and Biological Properties of Anions of Bivalent Transition Metal [Co(II) and Ni(II)] Complexes With Acylhydrazine Derived ONO Donor Schiff Bases

**DOI:** 10.1155/MBD.2000.133

**Published:** 2000

**Authors:** Zahid H. Chohan, M. A. Farooq, M. S. Iqbal

**Affiliations:** 1 Department of Chemistry Islamia University Bahawalpur Pakistan; 2 Himont Pharmaceutical and Chemical Laboratories Lahore Pakistan

## Abstract

Some acylhydrazine derived ONO donor Schiff bases and their Co(II) and Ni(II) complexes have been prepared having the same metal ion (cation) but different anions. These synthesized metal(II) complexes have been characterized on the basis of their elemental analyses, magnetic moment, molar conductance, and IR and electronic spectral data. All of the Schiff base ligands function as tridentates and the deprotonated enolic form is preferred for coordination. In order to evaluate the effect of anions on the bactericidal activity, these synthesized complexes, in comparison to the uncomplexed Schiff bases have been screened against bacterial species., *Escherichia coli*, *Staphylococcus aureus* and *Pseudomonas aeruginosa* and the results are reported.


### SYNTHESIS, CHARACTERIZATION AND BIOLOGICAL PROPERTIES OF ANIONS OF BIVALENT TRANSITION METAL [Co(II) AND Ni(II)] COMPLEXES WITH ACYLHYDRAZINE DERIVED ONO DONOR SCHIFF BASES

Zahid H. Chohan*[1], M. A. Farooq[1] and M. S. Iqbal[2]

[1]Department of Chemistry, Islamia University, Bahawalpur, Pakistan.
[2]Himont Pharmaceutical and Chemical Laboratories, Lahore, Pakistan

### ABSTRACT
Some acylhydrazine derived ONO donor Schiff bases and their Co(II) and Ni(II) complexes have been prepared having the same metal ion (cation) but different anions. These synthesized metal(II) complexes have been characterized on the basis of their elemental analyses, magnetic moment, molar conductance, and IR and electronic spectral data. All of the Schiff base ligands function as tridentates and the deprotonated enolic form is preferred for coordination. In order to evaluate the effect of anions on the bactericidal activity, these synthesized complexes, in comparison to the uncomplexed Schiff bases have been screened against bacterial species., *Escherichia coli, Staphylococcus aureus* and *Pseudomonas aeruginosa* and the results are reported.

### INTRODUCTION
Acylhydrazines have been shown to possess modest bacteriostatic properties[1,2] *in vitro* against many microorganisms. Recent studies have shown that these are also potent inhibitors of DNA synthesis in a variety of cultured human and rodent cells, and their metal complexes produce significant inhibition[3,4] against tumor growth. Their varied interesting ligational behavior[5-9] towards transition metal ions have also attracted the attention of many researchers[10-12] to explore their metalloorganic chemistry. Keeping in view all these aspects accordingly, some novel ONO donor acylhydrazines such as benzoylhydrazine, salicyloylhydrazine and nicotinoylhydrazine derived furanyl Schiff bases were previously synthesized[13] and the effect of metal ions on their antibacterial properties were reported. In the present studies, the same Schiff bases (Fig. 1) have been used for their complexation reactions with Co(II) and Ni(II) metal ions having different anions [$NO_3^-$, $SO_4^{2-}$, $C_2O_4^{2-}$ and $CH_3CO_2^-$] and the participating role of these anions on the antibacterial activity is further investigated and reported.

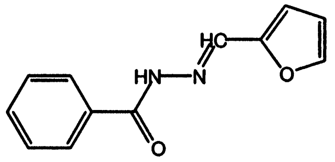

[L[1]]

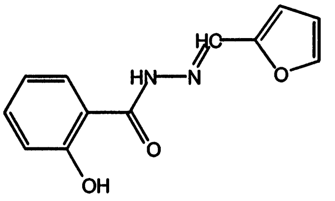

[L[2]]

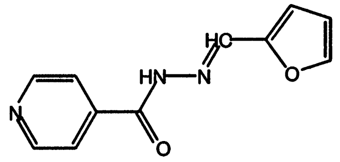

[L[3]]

Fig. 1. Proposed Structure of Schiff Bases

### EXPERIMENTAL
### Material and Methods
All chemicals and solvents used were of Analar grade. All metal(II) salts were used as sulphate, nitrate, acetate and oxalate. 2-Furanecarboxaldehyde, benzoylhydrazine, nicotinoylhydrazine and salicyloylhydrazine were obtained from Merck. IR spectra were recorded on a Philips Analytical PU 9800 FTIR spectrophotometer as KBr discs. UV-Visible spectra were obtained in DMF on a Hitachi U-2000 double-beam spectrophotometer. C, H and N analyses was carried out by Butterworth Laboratories Ltd. Conductance of the metal complexes was determined in DMF on a Hitachi YSI-32 model conductometer. Magnetic measurements were made on solid complexes using the Gouy method. Melting points were recorded on a Gallenkamp apparatus and are uncorrected.

**Preparation of Schiff bases**

The ligands were synthesized and fully characterized according to the same method reported[13] earlier.

**Preparation of Metal Complexes**

An ethanol solution (20 mL) of the appropriate metal(II) salt (0.001 M) was added to a magnetically stirred ethanol solution (15 mL) of the respective Schiff base (0.002 M). The mixture was refluxed for 2 h, then cooled to room temperature, filtered and reduced to nearly half its volume. The concentrated solution was left stand overnight at room temperature which resulted in the formation of a solid product. The product thus obtained was filtered, washed with ethanol, then with ether and dried. Crystallization in aqueous ethanol gave the desired metal (II) complexes.

**Antibacterial Studies**

The synthesized metal chelates, in comparison to the free Schiff bases were screened for their antibacterial activity against pathogenic bacterial species, *Escherichia coli, Staphylococcus aureus* and *Pseudomonas aeruginosa*. The paper disc diffusion method was adopted for the determination of antibacterial activity as reported[14-16] earlier.

### RESULTS AND DISCUSSION

**Physical Properties**

The Schiff bases (Fig. 1) were prepared by reacting equimolar amounts of 2-furanecarboxaldehyde with the respective benzoylhydrazine, salicyloylhydrazine or nicotinoylhydrazine in ethanol. The structures of these Schiff bases were established[13] with the help of their IR, $^1H$ NMR, $^{13}C$ NMR and microanalytical data. These synthesized Schiff bases are now used further for the preparation of its Co(II) and Ni(II) complexes having different anions as $NO_3^-$, $SO_4^{2-}$, $C_2O_4^{2-}$ and $CH_3CO_2^-$.

All of the metal complexes [(1)-(24)] (Table I) were prepared by the stoichiometric reaction of the respective metals as their sulphate, nitrate, acetate and oxalate, and Schiff bases in a molar ratio M:L = 1:2. All the complexes are intensely colored and stable crystalline solids, which decompose above 200°C without melting. The complexes are insoluble in common organic solvents like ethanol, methanol, chloroform or acetone. DMSO and DMF, however, dissolved all the complexes. Their melting behavior, solubility and crystalline nature suggest that they are monomers. Elemental analysis data (Table 1) indicated 1:2 (metal:ligand) stoichiometry. Molar conductance values of the soluble complexes in DMF show values (26-45 $ohm^{-1}cm^2mol^{-1}$) indicating[17] that they are all non-electrolytic in nature

**Model Studies**

Examination of the model of these Schiff bases illustrated in Fig. 2 show that in no case can the ligands exhibit quadridentate ONNO or bidentate ON or OO behavior. It is capable of exhibiting either tridentate monobasic (flexidentate keto-enol form) (Fig. 2) or neutral tridentate behavior. In its enol form, the ligand can function like an enol showing ONO donor by either deprotonation of the hydroxyl group of enol, ν(OH), nitrogen of hydrazine, ν(N-N), and heteroatom (O) of the furanyl group. The keto form however, acts as tridentate ONO donors showing coordination through the keto oxygen (C=O), azomethine nitrogen (CH=N) and the heteroatom (O).

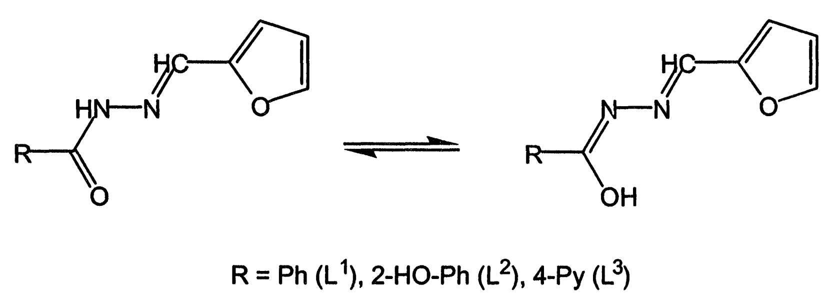

Fig. 2. Possible Flexidentate Keto-Enol Forms of Schiff Base

**Infrared Spectra**

a) The IR spectra were recorded in KBr and are depicted in Tables II with some tentative assignments of important characteristic bands. All the Schiff bases showed[13] the absence of bands at ~1735 and 3420 $cm^{-1}$ due to characteristic carbonyl ν(C=O) and $\nu(NH_2)$ stretching vibrations of the respective aldehydes and amines (starting materials). Instead, a new band at ~3240 $cm^{-1}$ and sharp bands at ~3215, 1700, 1635 and 1020 $cm^{-1}$ assigned[18,19] to amide-I [ν(CO-NH)], amide-II [ν(NH+CN)], azomethine [ν(C=N)] linkage and ν(N-N) modes appeared, respectively. It however, suggested that hydrazinoamine and aldehyde moieties of the starting reagents no longer exist and have been completely condensed and converted into their respective Schiff bases.

Table I. Physical and Analytical Data of the Metal(II) Complexes

| Complex/ Mol. Formula | M.P. (°C) | B.M. ($\mu_{eff}$) | Yield (%) | Calc. (Found) % C | H | N |
|---|---|---|---|---|---|---|
| (1) $[Co(L^1)_2(NO_3)_2]$ $C_{24}H_{18}CoN_4O_8$ [548.9] | 212-214 | 4.85 | 62 | 52.5 (52.7) | 3.3 (3.2) | 10.2 (10.1) |
| (2) $[Co(L^1)_2(SO_4)]$ $C_{24}H_{18}CoN_4O_8S$ [581.0] | 218-220 | 4.93 | 65 | 49.6 (49.9) | 3.1 (3.3) | 9.6 (9.3) |
| (3) $[Co(L^1)_2(C_2O_4)]$ $C_{26}H_{18}CoN_4O_8$ [572.9] | 231-233 | 4.87 | 60 | 54.5 (54.4) | 3.1 (3.3) | 9.8 (9.9) |
| (4) $[Co(L^1)_2(CH_3CO_2)_2]$ $C_{24}H_{24}CoN_4O_8$ [602.9] | 220-222 | 4.90 | 64 | 55.7 (55.5) | 4.0 (4.2) | 9.3 (9.6) |
| (5) $[Co(L^2)_2(NO_3)_2]$ $C_{24}H_{18}CoN_6O_{12}$ [640.9] | 218-220 | 4.92 | 60 | 44.9 (45.2) | 2.8 (2.5) | 13.1 (13.5) |
| (6) $[Co(L^2)_2(SO_4)]$ $C_{24}H_{18}CoN_4O_{10}S$ [613.0] | 225-227 | 4.89 | 58 | 47.0 (47.1) | 2.9 (2.7) | 9.1 (10.2) |
| (7) $[Co(L^2)_2(C_2O_4)]$ $C_{26}H_{18}CoN_4O_{10}$ [604.9] | 228-230 | 4.92 | 62 | 51.6 (51.9) | 3.0 (3.1) | 9.3 (9.0) |
| (8) $[Co(L^2)_2(CH_3CO_2)_2]$ $C_{28}H_{24}CoN_4O_{10}$ [634.9] | 222-224 | 4.90 | 65 | 52.9 (52.8) | 3.8 (3.6) | 8.8 (8.6) |
| (9) $[Co(L^3)_2(NO_3)_2]$ $C_{22}H_{16}CoN_8O_{10}$ [610.9] | 226-228 | 4.93 | 62 | 43.2 (43.0) | 2.6 (2.5) | 18.3 (18.5) |
| (10) $[Co(L^3)_2(SO_4)]$ $C_{22}H_{16}CoN_6O_8S$ [489.5] | 225-227 | 4.92 | 60 | 45.3 (45.5) | 2.7 (2.9) | 14.4 (14.2) |
| (11) $[Co(L^3)_2(C_2O_4)]$ $C_{24}H_{16}CoN_6O_8$ [574.9] | 230-232 | 4.89 | 64 | 50.1 (50.3) | 2.8 (2.9) | 14.6 (14.1) |
| (12) $[Co(L^3)_2(CH_3CO_2)_2]$ $C_{26}H_{22}CoN_6O_8$ [604.9] | 224-226 | 4.90 | 65 | 51.6 (51.5) | 3.6 (3.4) | 13.9 (13.7) |
| (13) $[Ni(L^1)_2(NO_3)_2]$ $C_{24}H_{18}NiN_4O_8$ [548.7] | 228-230 | 3.30 | 62 | 52.5 (52.3) | 3.3 (3.2) | 10.2 (10.3) |
| (14) $[Ni(L^1)_2(SO_4)]$ $C_{24}H_{18}NiN_4O_8S$ [580.8] | 225-227 | 3.52 | 60 | 49.6 (49.5) | 3.1 (3.6) | 9.6 (9.5) |
| (15) $[Ni(L^1)_2(C_2O_4)]$ $C_{26}H_{18}NiN_4O_8$ [572.7] | 230-232 | 3.20 | 65 | 54.5 (54.2) | 3.1 (3.2) | 9.8 (9.9) |
| (16) $[Ni(L^1)_2(CH_3CO_2)_2]$ $C_{28}H_{24}NiN_4O_8$ [602.7] | 232-234 | 3.70 | 65 | 55.7 (55.9) | 4.0 (3.8) | 9.3 (9.1) |
| (17) $[Ni(L^2)_2(NO_3)_2]$ $C_{24}H_{18}NiN_6O_{12}$ [640.7] | 228-230 | 3.25 | 64 | 45.0 (45.2) | 2.8 (3.0) | 13.1 (13.0) |
| (18) $[Ni(L^2)_2(SO_4)]$ $C_{24}H_{18}NiN_4O_{10}S$ [612.8] | 228-230 | 3.27 | 62 | 47.0 (47.2) | 2.9 (3.0) | 9.1 (9.5) |
| (19) $[Ni(L^2)_2(C_2O_4)]$ $C_{26}H_{18}NiN_4O_{10}$ [604.7] | 225-228 | 3.52 | 62 | 51.6 (51.8) | 3.0 (3.3) | 9.3 (9.1) |
| (20) $[Ni(L^2)_2(CH_3CO_2)_2]$ $C_{28}H_{24}NiN_4O_{10}$ [634.7] | 230-232 | 3.61 | 65 | 53.0 (53.1) | 3.8 (3.5) | 8.8 (8.5) |
| (21) $[Ni(L^3)_2(NO_3)_2]$ $C_{22}H_{16}NiN_8O_{10}$ [610.7] | 232-234 | 3.65 | 60 | 43.2 (43.5) | 2.6 (2.4) | 18.3 (18.5) |
| (22) $[Ni(L^3)_2(SO_4)]$ $C_{22}H_{16}NiN_6O_8S$ [582.8] | 230-232 | 3.45 | 62 | 45.3 (45.5) | 2.7 (2.5) | 14.4 (14.7) |
| (23) $[Ni(L^3)_2(C_2O_4)]$ $C_{24}H_{16}NiN_6O_8$ [574.7] | 232-234 | 3.50 | 65 | 50.1 (50.5) | 2.8 (2.7) | 14.6 (14.5) |
| (24) $[Ni(L^3)_2(CH_3CO_2)_2]$ $C_{26}H_{22}NiN_6O_8$ [604.7] | 235-237 | 3.55 | 64 | 51.6 (51.7) | 3.6 (3.7) | 13.9 (14.1) |

The comparison of the infrared spectra of the Schiff bases and their metal complexes indicated that the Schiff bases are coordinated to the metal atom by three sites, thus suggesting that the ligands act as a tridentate.

Table II. IR and UV-Visible Data of the Metal(II) Complexes

| No | IR ($cm^{-1}$) | $\lambda_{max}$ ($cm^{-1}$) |
|---|---|---|
| (1) | 3215 (s, NH), 1625 (s, CH=N), 1245 (s, C-O), 1025 (m, N-N), 455 (m, M-O) | 8545, 17785, 20420 |
| (2) | 3210 (s, NH), 1625 (m, CH=N), 1240 (s, C-O), 1030 (m, N-N), 460 (m, M-O) | 8610, 17815, 20485 |
| (3) | 3220 (s, NH), 1630 (s, CH=N), 1240 (m, C-O), 1030 (m, N-N), 455 (m, M-O) | 8585, 17825, 20445 |
| (4) | 3210 (s, NH), 1625 (s, CH=N), 1245 (s, C-O), 1025 (m, N-N), 455 (m, M-O) | 8570, 17810, 20460 |
| (5) | 3210 (s, NH), 1625 (m, CH=N), 1245 (s, C-O), 1025 (m, N-N), 455 (m, M-O) | 8585, 17795, 20475 |
| (6) | 3220 (s, NH), 1630 (m, CH=N), 1245 (m, C-O), 1030 (m, N-N), 455 (m, M-O) | 8555, 17785, 20480 |
| (7) | 3210 (s, NH), 1625 (s, CH=N), 1240 (s, C-O), 1025 (m, N-N), 455 (m, M-O) | 8550, 17795, 20455 |
| (8) | 3210 (s, NH), 1630 (s, CH=N), 1240 (m, C-O), 1025 (m, N-N), 455 (m, M-O) | 8555, 17780, 20480 |
| (9) | 3210 (s, NH), 1625 (s, CH=N), 1245 (s, C-O), 1025 (m, N-N), 460 (m, M-O) | 8592, 17815, 20485 |
| (10) | 3215 (s, NH), 1625 (s, CH=N), 1245 (s, C-O), 1025 (m, N-N), 455 (m, M-O) | 8585, 17795, 20475 |
| (11) | 3210 (s, NH), 1625 (m, CH=N), 1245 (m, C-O), 1030 (m, N-N), 455 (m, M-O) | 8605, 17790, 20455 |
| (12) | 3220 (s, NH), 1630 (m, CH=N), 1240 (s, C-O), 1025 (m, N-N), 455 (m, M-O) | 8555, 17810, 20425 |
| (13) | 3210 (s, NH), 1625 (s, CH=N), 1240 (s, C-O), 1025 (m, N-N), 455 (m, M-O) | 10270, 19055, 26880 |
| (14) | 3210 (s, NH), 1625 (s, CH=N), 1240 (s, C-O), 1025 (m, N-N), 455 (m, M-O) | 10320, 19145, 27085 |
| (15) | 3210 (s, NH), 1625 (s, CH=N), 1240 (s, C-O), 1025 (m, N-N), 455 (m, M-O) | 10285, 19105, 26885 |
| (16) | 3210 (s, NH), 1625 (s, CH=N), 1240 (s, C-O), 1025 (m, N-N), 455 (m, M-O) | 10315, 19135, 26990 |
| (17) | 3210 (s, NH), 1625 (s, CH=N), 1240 (s, C-O), 1025 (m, N-N), 455 (m, M-O) | 10310, 19120, 26895 |
| (18) | 3210 (s, NH), 1625 (s, CH=N), 1240 (s, C-O), 1025 (m, N-N), 455 (m, M-O) | 10292, 19140, 26900 |
| (19) | 3210 (s, NH), 1625 (s, CH=N), 1240 (s, C-O), 1025 (m, N-N), 455 (m, M-O) | 10290, 19135, 26915 |
| (20) | 3210 (s, NH), 1625 (s, CH=N), 1240 (s, C-O), 1025 (m, N-N), 455 (m, M-O) | 10300, 19120, 26915 |
| (21) | 3210 (s, NH), 1625 (s, CH=N), 1240 (s, C-O), 1025 (m, N-N), 455 (m, M-O) | 10310, 19130, 26885 |
| (22) | 3210 (s, NH), 1625 (s, CH=N), 1240 (s, C-O), 1025 (m, N-N), 455 (m, M-O) | 10285, 19145, 26895 |
| (23) | 3210 (s, NH), 1625 (s, CH=N), 1240 (s, C-O), 1025 (m, N-N), 455 (m, M-O) | 10275, 19110, 26880 |
| (24) | 3210 (s, NH), 1625 (s, CH=N), 1240 (s, C-O), 1025 (m, N-N), 455 (m, M-O) | 10310, 19135, 26895 |

s = sharp, m = medium b) The band appearing at ~3240 $cm^{-1}$ due to amide-I band was substantially shifted to lower frequency (20-30 $cm^{-1}$) relative to the free ligand values and the bands due to ν(C=O) were completely missing in the spectra of the complexes, suggesting[20] enolization of the Schiff bases on complexation. This is also supported by the fact that no band for ν(OH) in the spectra of the Schiff bases and also in the complexes is observed. Instead, a band due to ν(C-O) at about ~1245 $cm^{-1}$ was observed for all the title complexes, which supports the observation of their enolization during coordination. These facts suggest that the Schiff bases remain in the keto form in the solid state but in solution, both the keto and enol forms remain in equilibrium[21] (Fig. 2) and during complexation, deprotonation occurs from the enol form. This coordination through the deprotonated enolized oxygen results in a ν(NCO) band manifested in the region at ~1245-1250 $cm^{-1}$.

c) The amide-II band was also split, displaced to higher frequency and reduced in intensity. Shift to the higher frequency (5-10 $cm^{-1}$) of the ν(N-N) band at ~1025 $cm^{-1}$ and its splitting indicated[22] coordination of the azomethine nitrogen. Moreover, the lower-frequency shift (5-10 $cm^{-1}$) of the band due to the azomethine ν(CH=N) linkage at ~1635 $cm^{-1}$ also indicated involvement of azomethine group in coordination.

The appearance of new weak low-frequency bands at ~455 $cm^{-1}$ were assigned[23] to metal-oxygen ν(M-O). These bands were only observable in the spectra of the metal complexes and not in the spectra of their Schiff bases which in turn confirmed the participation of the heteroatoms (O) in the coordination.

**Magnetic Moments and Electronic Spectra**

UV-Visible spectral bands of the complexes are recorded in Table III. The cobalt(II) complexes show magnetic moment values of 4.85-4.93 B.M. at room temperature. These high values of the magnetic moments and the stoichiometries suggest[24,25] a coordination number of six for the central cobalt(II) ion and attaining an octahedral geometry. The CHN analyses also support this inference. The electronic spectra of these complexes are also consistent with their octahedral environment around the cobalt(II) ion. The spectra display bands at ~8,545-8,610, ~17,785-17,815 and ~20,420-20,485 $cm^{-1}$ attributed to $^4T_{1g}$ (F) → $^4T_{2g}$ (F)($V_1$), $^4T_{1g}$ (F) → $^4A_{2g}$ (F)($V_2$) and $^4T_{1g}$ → $^4T_{1g}$ (P)($V_3$) transitions, respectively, in a high-spin octahedral geometry[26,27]. The electronic spectra of the nickel(II) complexes exhibited three typical absorption bands at ~10,270-10320, 19055-19145 and ~26,880-27085 $cm^{-1}$, corresponding to the transitions $^3A_{2g}$ → $^3T_{2g}$ ($V_1$), $^3A_{2g}$ → $^3T_{1g}$ (F)($V_2$), and $^3A_{2g}$ → $^3T_{2g}$ (F)($V_3$), respectively, characteristic for their octahedral environment[28-30]. Also, the values of the magnetic moment (3.2-3.7 B.M.) may be taken as additional evidence[31,32] for their octahedral structure.

On the basis of the above observations, it is tentatively suggested that all of the complexes show an octahedral geometry (Fig. 3) in which the two ligands act as tridentates. These possibly accommodate themselves around the metal atom in such a way that a stable chelate ring is formed giving in turn, stability to the formed metal complexes.

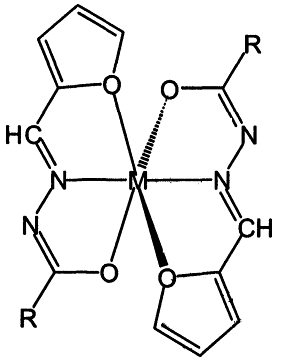

R = Ph; R = 2-HOPh, 4-Py

M = Co(II), Ni(II)

Fig. 3. Proposed Structure of the Metal(II) Complexes

**Antibacterial Properties**

The title Schiff bases in caomparison to their metal(II) chelates having the same metal atom but different anions were evaluated for their antibacterial activity against the standard bacterial strains of *Escherichia coli (a),), Staphylococcus aureus (b)* and *Pseudomonas aeruginosa (c)* The compounds were tested at a concentration of 30 μg/0.01 mL in DMF solution using the paper disc diffusion method reported[33,34] as earlier. The susceptibility zones were measured in mm and the results are reproduced in Table III. The susceptibility zones were the clear zones around the discs, which were measured in mm. All the Schiff bases and their complexes individually were found to be biologically active showing various degrees of inhibitory effects on the growth of the tested bacterial species. The antibacterial results evidently show that the activity of the Schiff bases became more upon coordination to the metal atoms. But, when the same metal complex having different anions was individually screened the degree of bacteriostatic activity also changed. From the obtained data as reproduced in Table III, it was generally observed that the order of potency in comparison to the metal complexes having chloride anions evaluated and reported earlier and the results of the present studies against the same tested bacterial strains under the same conditions were found to follow the order as $NO_3 > C_2O_4 > CH_3CO_2 > Cl > SO_4$.

Table III. Antibacterial Activity Data of the Schiff bases and its Metal(II) Complexes

| Schiff base/ Complex | Microbial Species a | b | c |
|---|---|---|---|
| $HL^1$ | + | ++ | ++ |
| $HL^2$ | ++ | + | + |
| $HL^3$ | ++ | + | ++ |
| (1) | +++ | ++ | +++ |
| (2) | +++ | +++ | ++ |
| (3) | +++ | ++ | ++++ |
| (4) | +++ | +++ | +++ |
| (5) | ++++ | +++ | +++ |
| (6) | +++ | +++ | ++ |
| (7) | ++++ | ++ | +++ |
| (8) | ++ | +++ | ++++ |
| (9) | +++ | +++ | +++ |
| (10) | +++ | +++ | +++ |
| (11) | +++ | +++ | +++ |
| (12) | +++ | ++++ | ++ |
| (13) | ++ | ++ | +++ |
| (14) | +++ | +++ | +++ |
| (15) | +++ | ++++ | ++ |
| (16) | ++ | +++ | +++ |
| (17) | ++++ | +++ | ++ |
| (18) | +++ | +++ | ++ |
| (19) | +++ | +++ | +++ |
| (20) | +++ | +++ | ++ |
| (21) | +++ | ++++ | +++ |
| (22) | +++ | +++ | +++ |
| (23) | ++++ | +++ | ++ |
| (24) | +++ | +++ | +++ |

a= *Escherichia coli*, b= *Staphylococcus aureus*,
c= *Pseudomonas aeruginosa*
Inhibition zone diameter mm (% inhibition): +, 6-10 (27-45 %); ++, 10-14 (45-64 %); +++, 14-18 (64-82 %); ++++, 18-22 (82-100 %). Percent inhibition values are relative to inhibition zone (22 mm) with 100 % inhibition.

On the basis of these results, it is claimed that different anions which stay outside the coordination sphere of the complex as counter ions also effect the biological behavior of the metals (cations). It is suspected that possible factors, such as solubility, conductivity, dipole moment and cell permeability mechanism that may cause an effect to induce more potency/activity in these metal chelates. However, our *in vivo* studies are in progress which may help to establish this mechanism.

### ACKNOWLEDGEMENT
The author gratefully acknowledges the help of the Department of Microbiology, Qaid-e-Azam Medical College, Bahawalpur, in performing the antibacterial studies.

### REFERENCES
1. H. A. Offe, W. Siefken and G. Domagk, *Z. Naturforsch*, **7**, 446 (1952).
2. J. R. Dimmock, G. B. Baker and W. G. Taylor, *Can. J. Pharm. Sci*, **7**, 100 (1972).
3. D. K. Johnson, T. B. Murphy, N. J. Rose, W. H. Goodwin and L. Pickart, *Inorg. Chim. Acta*, **67**, 159 (1982).

4. L. Pickart, W. H. Goodwin, W. Burgua, T. B. Murphy and D. K. Johnson, *Biochem. Pharmacol*, **32**, 3868 (1983).
5. C. Pellizi, G. Pellizi and F. Vitali, *J. Chem. Soc (Dalton Trans),* 177 (1987).
6. T. M. Amminabhavi, N. S. Biradar and W. E. Rudziuk, *Inorg. Chim. Acta*, **78**, 107 (1983).
7. T. B. Murphy, D. K. Johnson, N. J. Rose, A. Aruffo and V. Schomaker, *Inorg. Chim. Acta,* **66**, L-67 (1982).
8. R. Haran, J. Gairin and G. Commenges, *Inorg. Chim. Acta*, **63**, 46 (1980).
9. C. Pellizi, G. Pellizi, G. Predieri and S. Resola, *J. Chem. Soc.(Dalton Trans),* 1349 (1982).
10. L. El. Sayed and M. F. Iskander, *J. Inorg. Nucl. Chem*, **33**, 435 (1971).
11. M. F. Iskander and L. El. Sayed, *Inorg. Chim. Acta*, **16**, 147 (1976).
12. M. F. Iskander and A. M. El. Aggan, *Inorg. Chim. Acta*, **14**, 167 (1975).
13. Z. H. Chohan, *Synth. React. Inorg. Met.-Org. Chem*, (Accepted for publication)
14. Z. H. Chohan, *Metal-Based Drugs*, **6**, 187 (1999).
15. Z. H. Chohan and S. Kausar, *Metal-Based Drugs*, **7**, 17 (2000).
16. Z. H. Chohan and S. K. A. Sherazi, *Synth. React. Inorg. Met.-Org. Chem*, **29**, 105 (1999).
17. W. J. Geary, *Coord. Chem. Rev*, **7**, 81 (1971).
18. K. Burger, I. Ruff and F. Ruff, *J. Inorg. Nucl. Chem*, **27**, 179 (1965).
19. M. Mashima, *Bull. Chem. Soc. Japan*, **35**, 1882 (1962).
20. S. R. Patil, U. N. Kantak and D. N. Sen, *Inorg. Chim. Acta*, **63**, 261 (1982).
21. K. Dey and D. Bandyopadhyay, *Trans. Metal. Chem*, **16**, 267 (1991).
22. M. Yongxiang, Z. Zhengzhi, M. Yun and Z. Gang, *Inorg. Chim. Acta*, **165**, 185 (1989).
23. K. Nakamoto, "*Infrared Spectra of Inorganic and Coordination Compounds*", 2nd Edn., Wiley Interscience, New York (1970).
24. M. D. Glick and R. N. Lintvedt, *Prog. Inorg. Chem*, **21**, 233 (1976).
25. C. J. Baulhausen, "*An Introduction to Ligand Field*", Mc Graw Hill, New York (1962).
26. A. B. P. Lever, "*Inorganic Electronic Spectroscopy*", 2nd Ed, Elsevier, New York (1984).
27. A. D. Liehr, *J. Phys. Chem*, **67**, 1314 (1967).
28. S. Chandra, *Polyhedron*, **4**, 663 (1985).
29. B. N. Figgis and J. Lewis, *Prog. Inorg. Chem*, Ed. F. A. Cotton, Interscience, **6**, 37 (1964).
30. R. C. Agarwal and C. Vallabhaneve, *Trans. Met. Chem*, **3**, 309 (1978).
31. A. B. P. Lever, Inorg. Chem, 4, 763 (1965)
32. V. B. Rama, D. D. Singh, P. Singh and M. Teotia, *Trans. Met. Chem*, **6**, 36 (1981).
33. Z. H. Chohan, S. K. A. Sherazi and M. S. Iqbal, *Metal-Based Drugs*., **5**, 347 (1998).
34. Z. H. Chohan, M. Praveen and A. Ghaffar, *Synth. React. Inorg. Met.-Org. Chem*, **28**, 1673 (1998).

**Received: April 7, 2000 - Accepted: May 9, 2000 -**
**Received in publishable format: May 10, 2000**

